# The role of MRI after neochemoradiotherapy in predicting pathological tumor regression grade and clinical outcome in patients with locally advanced rectal adenocarcinoma

**DOI:** 10.3389/fonc.2023.1118518

**Published:** 2023-06-12

**Authors:** Shaoqing Niu, Yan Chen, Fang Peng, Jie Wen, Jianqi Xiong, Zhuangzhuang Yang, Jianjun Peng, Yong Bao, Li Ding

**Affiliations:** ^1^Department of Radiation Oncology, the First Affiliated Hospital, Sun Yat-sen University, Guangzhou, China; ^2^Department of Radiology, the First Affiliated Hospital, Sun Yat-sen University, Guangzhou, China; ^3^Department of Interventional Oncology, the First Affiliated Hospital, Sun Yat-sen University, Guangzhou, China; ^4^Gastrointestinal Surgery Center, the First Affiliated Hospital, Sun Yat-sen University, Guangzhou, China; ^5^Department of Pathology, the First Affiliated Hospital, Sun Yat-sen University, Guangzhou, China

**Keywords:** rectal cancer, neoadjuvant therapy, magnetic resonance imaging, tumor regression grade, prognosis

## Abstract

**Objective:**

To evaluate the predictive value of tumor regression grade assessed by MRI (mr-TRG) after neoadjuvant chemoradiotherapy (neo-CRT) for postoperative pathological TRG (pTRG) and prognosis in patients with locally advanced rectal adenocarcinoma (LARC).

**Materials and methods:**

This was a retrospective study from a single center experience. The patients who were diagnosed with LARC and received neo-CRT in our department between January 2016 and July 2021 were enrolled. The agreement between mrTRG and pTRG was assessed with the weighted κ test. Overall survival (OS), progress-free survival (PFS), local recurrence-free survival (LRFS), and distant metastasis-free survival (DMFS) were calculated by Kaplan-Meier analysis and log-rank test.

**Results:**

From January 2016 to July 2021, 121 LARC patients received neo-CRT in our department. Among them, 54 patients had complete clinical data, including MRI of pre- and post-neo-CRT, postoperative tumor samples, and follow-up. The median follow-up time was 34.6 months (range: 4.4-70.6 months). The estimated 3-year OS, PFS, LRFS and DMFS were 78.5%, 70.7%, 89.0%, and 75.2%, respectively. The median time from the completion of neo-CRT to preoperative MRI and surgery was 7.1 weeks and 9.7 weeks, respectively. Out of 54 patients, 5 patients achieved mrTRG1 (9.3%), 37 achieved mrTRG2 (68.5%), 8 achieved mrTRG3 (14.8%), 4 achieved mrTRG4 (7.4%), and no patient achieved mrTRG5 after neo-CRT. Regarding pTRG, 12 patients achieved pTRG0 (22.2%), 10 achieved pTRG1 (18.5%), 26 achieved pTRG2 (48.1%), and 6 achieved pTRG3 (11.1%). The agreement between three-tier mrTRG (mrTRG1 vs. mrTRG2-3 vs. mrTRG4-5) and pTRG (pTRG0 vs. pTRG1-2 vs. pTRG3) was fair (weighted kappa=0.287). In a dichotomous classification, the agreement between mrTRG(mrTRG1 vs. mrTRG2-5)and pTRG(pTRG0 vs. pTRG1-3) also resulted in fair agreement (weighted kappa=0.391). The sensitivity, specificity, positive, and negative predictive values of favorable mrTRG (mrTRG 1-2) for pathological complete response (PCR) were 75.0%, 21.4%, 21.4%, and 75.0%, respectively. In univariate analysis, favorable mrTRG (mrTRG1-2) and downstaging N were significantly associated with better OS, while favorable mrTRG (mrTRG1-2), downstaging T, and downstaging N were significantly associated with superior PFS (*p*<0.05). In multivariate analysis, downstaging N was an independent prognostic factor for OS. Meanwhile, downstaging T and downstaging N remained independent prognostic factors for PFS.

**Conclusions:**

Although the consistency between mrTRG and pTRG is only fair, favorable mrTRG after neo-CRT may be used as a potential prognostic factor for LARC patients.

## Introduction

Colorectal cancer is the third most common type of cancer and the second leading cause of cancer death according to GLOBOCAN 2020 estimates (1). Different treatment strategies were adopted for different tumor-node-metastasis (TNM) stage diseases combined with clinical features, such as the status of circumferential resection margin (CRM) and extramural venous invasion (EMVI) (2). According to the latest NCCN guidelines, neoadjuvant chemoradiotherapy (neo-CRT) followed by surgery and postoperative chemotherapy (ChT) is the standard care for patients with stage II-III rectal cancer (2).

In the whole process of diagnosis and treatment, pelvic MR and postoperative pathological results play a fatal role in making appropriate treatment decisions for locally advanced rectal adenocarcinoma (LARC) patients. Taking advantage of superior soft-tissue contrast and the ability to allow multiplanar imaging and functional evaluation, MRI is not only considered to be the gold standard of rectal cancer staging but also the best way to assess response to neo-CRT and predict prognosis (3–5). Mandard, Dworak, the American Joint Committee on Cancer (AJCC) and the College of American Pathologists (CAP) created different pathology tumor regression grade (pTRG) systems to evaluate different tumor responses to neo-CRT, which were indicated to be effective and prognostic factors in future studies (6–10). Then, the MERCURY study group established an MRI-assessed tumor regression grade (mr-TRG) system that was analogous to pTRG (5).

However, there was no consistent result of the agreement between mrTRG and pTRG (11, 12). Here, we enrolled 54 LARC patients who received neo-CRT and surgery. In addition to complete routine clinicopathological data, all of them had MRI examination pre- and post- neo-CRT, and operative specimens. The responses of each patient were assessed by MRI after neo-CRT (mrTRG) and by postsurgical histopathologic specimens (pTRG). This study aimed to investigate the consistency of mrTRG and pTRG in these pTRG-defined patients, and to evaluate the predictive value of mr-TRG for prognosis. Furthermore, we hope to provide more valuable information for LARC patients before surgery, and even give some patients who were assessed with favorable mrTRG the opportunity to choose “watch and wait”, which is an organ preservation treatment strategy (13).

## Materials and methods

### Patients

This was an observational study approved by our institutional medical ethics committee (No [2021].125). From January 2016 to July 2021, 121 LARC patients received neo-CRT at Department of Radiotherapy of the First Affiliated Hospital, Sun Yat-sen University. Before treatment, written informed consent was obtained from all patients. All patients were confirmed histologically as adenocarcinoma. Imaging diagnoses of them were stage II or III disease by pelvic MRI, chest/abdominal CT with contrast, and endorectal ultrasound.

### Treatment details

The treatment strategies of all patients were managed by the gastrointestinal center multidisciplinary team (MDT). All patients received long-course radiotherapy (LCRT) with concurrent ChT and surgery. Radiotherapy (RT) was delivered with volume modulated arc therapy (VMAT). Gross tumor volume (GTV) was defined as the primary tumor (GTVp) and positive lymph nodes (GTVn). Clinical target volume (CTV) included the GTV plus areas at risk for microscopic spread from the primary tumor and at-risk nodal areas (14). The prescribed doses delivered to GTVp and GTVn were 50 Gy for 43 patients, 52.5 Gy for 10 patients, 60 Gy for one patient respectively, and the doses delivered to CTV were 45 Gy for 45 patients, 46 Gy for 9 patients respectively, all delivered in 25 daily fractions. The surgical procedures include Dixon, Miles, Parks, Hartmann and local excision.

The concurrent ChT regimens with RT included CapeOx (Oxaliplatin 130 mg/m^2^ on Day 1 + Capecitabine 1000 mg/m^2^ twice daily for 14 days, repeated for 3 weeks)for 28 patients, Capecitabine (825mg/m^2^ twice daily for 5 days a week) for 22 patients, and mFOLFOX (Oxaliplatin 85 mg/m^2^ on Day 1, leucovorin 400 mg/m^2^ on Day 1, 5-Fu 400 mg/m^2^ bolus on Day 1, followed by 1200 mg/m^2^/day for 2 days, over 46-48 hours continuous infusion, repeated for 2 weeks) for 4 patients. The adjuvant ChT regimens included CapeOx for 29 patients and mFOLFOX for 4 patients.

### MRI examination and evaluation

Appropriate (20-80 mL) ultrasound gel was used to provide enhanced depiction of the tumor, except for patients with low or large rectal tumors. Before imaging, 20 mg of raceanisodamine hydrochloride was intramuscularly injected to decrease intestinal peristalsis artefacts. All rectal MR images were performed using a 3.0 T MR scanner (Magnetom Verio, Siemens Healthcare, Erlangen, Germany) with a 6-channel phased-array surface coil. All patients were imaged in the supine position and oriented feet-first. The imaging protocols comprised (a) axial turbo spin-echo T2-weithed imaging (T2WI); (b) high-spatial-resolution turbo spin-echo T2WI in sagittal, coronal and oblique axial planes with the oblique axial plane perpendicular to the tumor base; and (c) axial diffusion-weighted imaging (DWI) with b factors of 0 and 1000 s/mm^2^ using a single-shot echo-planner imaging sequence. Detailed protocols are listed in **Table 1**.

**Table 1:** MRI protocols for Rectal Cancer.

Parameters	Axial T2WI	Sagittal T2WI	Coronal T2WI	Oblique axial T2WI	Axial DWI
TR/TE (ms)	3000/87	3000/87	4000/77	3000/84	3800/74.4
Slice thickness(mm)	5	3	3	3	6
Distance factor (%)	20	0	0	0	20
Slices	25	19	25	24	21
FOV(mm^2^)	260×260	180×180	220×220	180×180	300×245
Voxel size (mm^3^)	0.8× 0.7×5.0	0.7× 0.6×3.0	0.7× 0.6×3.0	0.6× 0.6×3.0	2.7× 2.7×6.0
Time acquisition	2 min 54 s	2 min 30 s	2 min 52 s	3 min 18 s	6 min 1 s

TR, repetition time; TE, echo time; FOV, field of view; T2WI, T2-weighted imaging; DWI, diffusion-weighted imaging.

Two radiologists experienced in rectal MRI (6 and 5 years) independently reviewed the paired MR images (pre- and post-neo-CRT) without knowledge of the postoperative histopathological results. For a consensus or majority decision, discrepancies were resolved by a third radiologist with more than 20 years of experience in rectal MRI.

**Figure 1:**
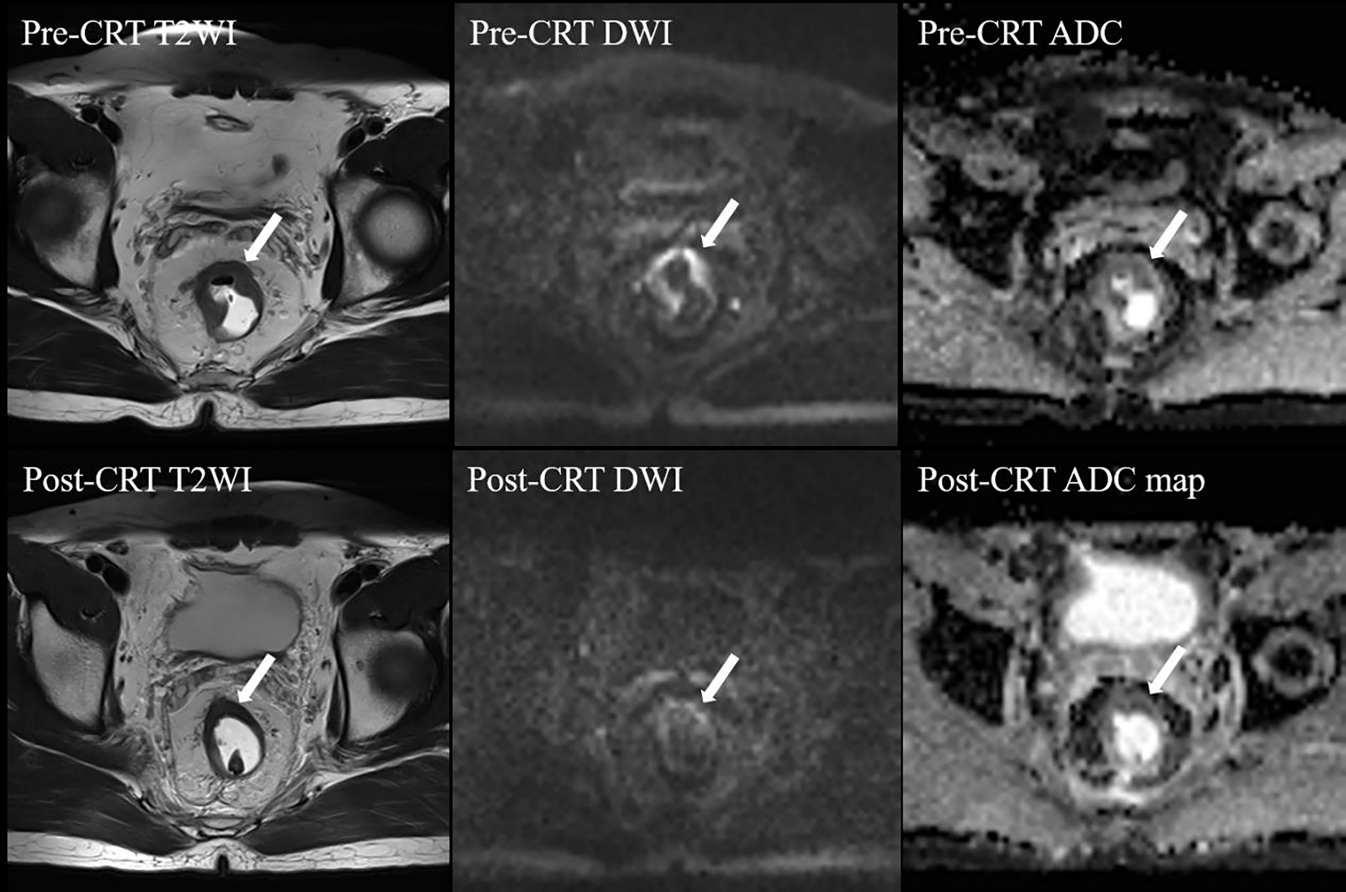
MRI tumor regression grade 2 in a 43-year-old man after neo-CRT. Post-neo-CRT T2WI shows a remarkable decrease in the tumor size with the remaining hypointense “fibrotic” thickening of the wall without visible tumor signal, whereas high *b* value post-CRT-DWI shows linear high signal intensity at the tumor bed, with low signal intensity on post-CRT-ADC map, indicating residual tumor. CRT, chemoradiotherapy; T2WI, T2-weighed imaging; DWI, diffusion-weighted imaging; ADC, apparent diffusion coefficient. The white arrows indicate tumor.

**Figure 2:**
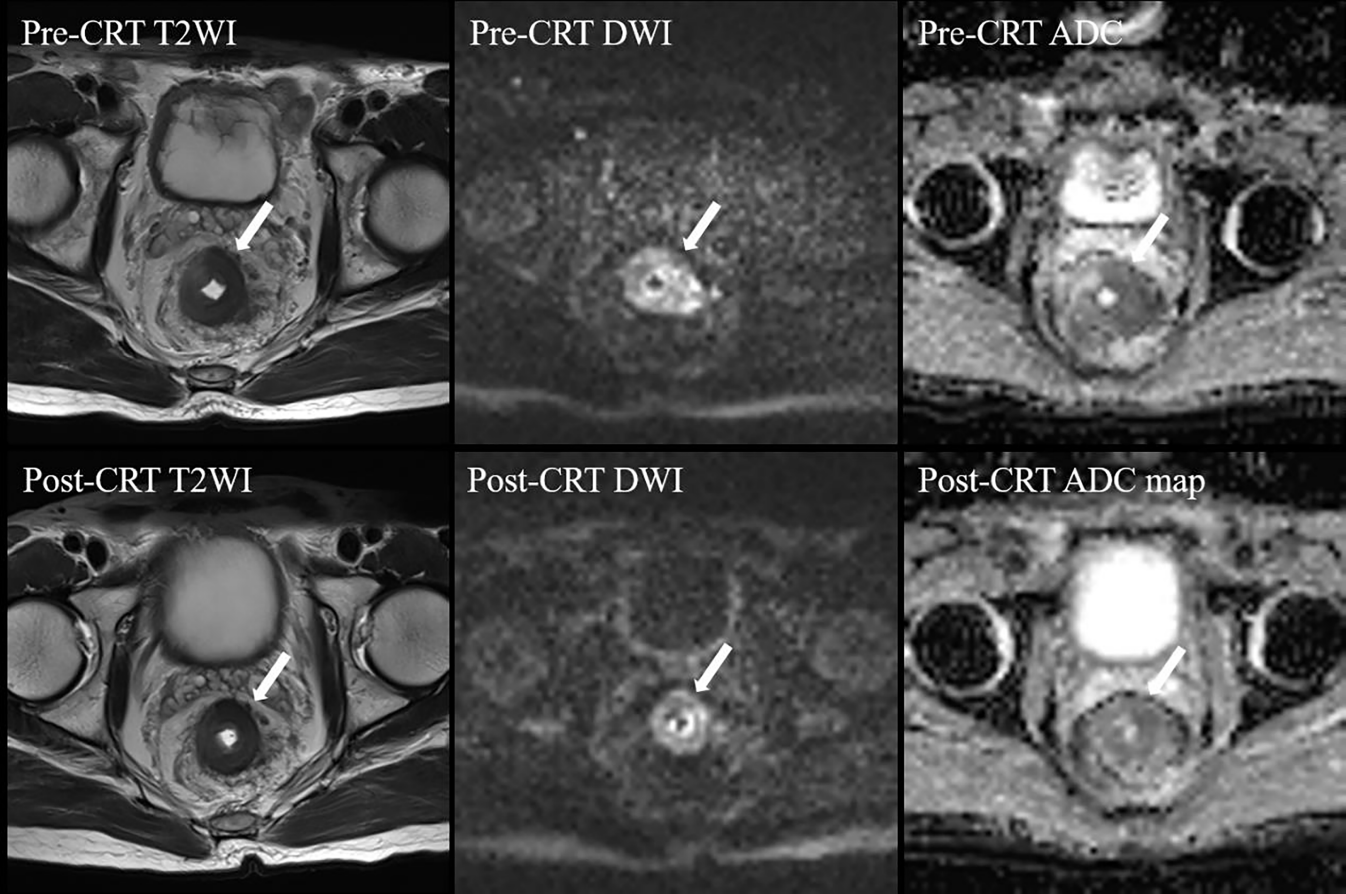
MRI tumor regression grade 4 in a 59-year-old man after CRT. Post-CRT T2WI shows a slight reduction (< 50%) in the tumor size with a majority of intermediate signal intensity. On high b value post-CRT-DWI, a thick layer of diffusion restriction was apparent at the tumor bed. CRT, chemoradiotherapy; T2WI, T2-weighed imaging; DWI, diffusion-weighted imaging; ADC, apparent diffusion coefficient.

A semiquantitative MRI-based tumor regression grade has been implemented (3) (**Supplementary materials**). We combined T2WI and DWI to assess the relative proportions of residual tumor and the degree of morphologic changes such as fibrosis and mucin production on post-neo-CRT MR images (15, 16). On T2WI, tumor fibrosis demonstrates a signal intensity similar to that of the normal muscularis propria, mucin production within a treated tumor manifests as an interval increase in signal intensity, and residual tumor demonstrates a more intermediate signal intensity similar to that on pretreatment MR images. A hyperintense signal on high-b-value (1000 s/mm^2^) DWI at the former tumor location, with low signal intensity on the apparent diffusion coefficient map, was considered to be tumor signal. When there was a discrepancy between two image sets, it was resolved through a complementary approach. For example, confusingly high signal intensity of a lesion on DWI that might have been caused by mucinous change or artifacts was interpreted on T2WI and an apparent diffusion coefficient map. In addition, it should give priority to DWI when ambiguously intermediate high signal intensity of a lesion on T2WI that was difficult to decide residual tumor or radiation fibrosis. Two patients who were assessed as mrTRG2 and mrTRG4 after neo-CRT are presented in **Figure 1**, **2**, respectively.

### Pathological evaluation

**Figure 3:**
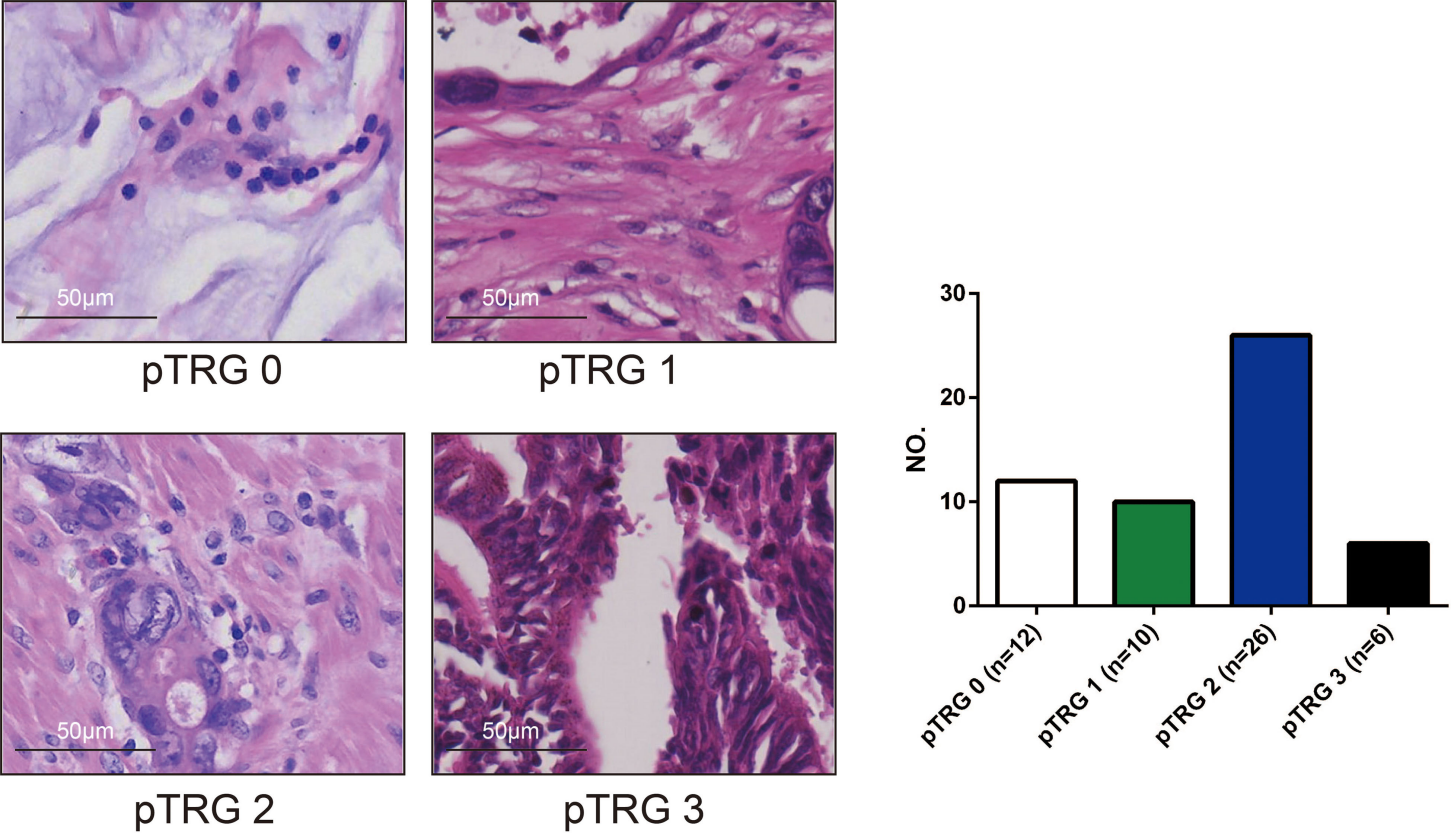
The pathological tumour regression (pTRG) assessment results (photomicrograph H and E, 400x). There were 12 patients with pTRG grade 0, 10 patients with pTRG grade 1, 26 patients with pTRG grade 2, and 6 patients with pTRG grade 3, respectively.

Formalin fixation and paraffin-embedding (FFPE) tissue sections were cut into 5 μm thick slices and fixed in 4% paraformaldehyde PFA for assessment. Subsequently, the slices were used to perform haematoxylin-eosin (HE) staining. The slides were stained following the HE staining kit (Solarbio, G1120) protocol and observed by microscopy. The specimens were examined and analysed by a pathologist with 10 years of experience and were further reviewed by a dedicated gastrointestinal pathologist, both of whom were blinded to the MRI data. The pTRG-based tumor regression grade assessment system recommended by the AJCC Cancer Staging Manual, Eighth Edition and the CAP Guidelines (17, 18) was implemented in this study (**Figure 3**) (**Supplementary material**). Pathological complete response (PCR) was defined as the absence of viable tumor cells in the primary tumor and lymph nodes.

### Follow-up

The follow-up visits were performed every 3 months during the first 2 years after treatment, every 6 months in the subsequent 3 years, and then yearly thereafter. Patients were followed up by telephone until the last visit on March 10, 2022 or death.

### Statistical analysis

The primary end point is the agreement between mrTRG and pTRG. The strength of agreement was assessed using the weighted kappa test. Kappa values were assessed as follows: 0.81-1.00, excellent agreement; 0.61-0.80, good agreement; 0.41-0.60, moderate agreement; 0.21-0.40, fair agreement; and 0.00-0.20, poor agreement. Dichotomous classification for mrTRG (mrTRG1-2 vs. mrTRG 3-5) and pTRG (pTRG 0 vs. pTRG 1-3) was performed to assess the ability of mrTRG to identify PCR by calculating sensitivity, specificity, and positive and negative predictive values.

The secondary end points were the overall survival (OS), local-regional recurrence-free survival (LRFS), progression-free survival (PFS), and distant metastasis-free survival (DMFS) of the enrolled patients. The Kaplan-Meier method and log-rank test were used to assess the differences between favorable (mrTRG1-2) and unfavorable (mrTRG 3-5) patients. *P* values of less than 0.05 with two sides were considered statistically significant. Survival was analyzed with the log-rank test by SPSS software (SPSS. Inc., Version 25, Chicago, IL).

## Results

### Clinicopathological characteristics

Except for the patients with missing MRI or post surgery specimens or those lost to follow-up, a total of 54 patients with complete clinical data, including MRI information (before and after neo-CRT), postoperative tumor samples, and follow-up, were enrolled in this retrospective study. There were 40 males and 14 females, and the median age at diagnosis was 55 years old (range: 27-74 years). There were 2 patient with stage II disease and 52 patients with stage III disease. There were 42 patients with positive CRM, and 12 patients with negative CRM. There were 38 patients with positive EMVI, 16 patients with negative EMVI. Thirteen patients were diagnosed as peritoneal reflection involved before treatment. The tumor locations included 3 in the upper rectum, 35 in the middle rectum, and 16 in the lower rectum. All clinicopathological characteristics were summarized in **Table 2**.

**Table 2:** Baseline characteristics and predictive value for prognosis.

Characteristic	N	(%)	OS		PFS		LRFS	
			3-y rate (%)	*P*	3-y rate (%)	*P*	3-y rate (%)	*P*
Age								
≤50y	20	37.0	86.1	0.124	70.4	0.702	95.0	0.843
>50y	34	63.0	73.3		72.0		96.4	
Meidan: 55 years old, rang (27-74)								
Gender								
Male	40	74.1	71.6	0.215	67.5	0.398	94.3	0.404
Female	14	25.9	100		83.6		100	
Tumor location*								
Upper	3	5.6	100	0.318	66.7	0.672	100	0.500
Middle	35	64.8	74.0		66.5		92.8	
Lower	16	29.6	82.0		79.8		100	
cT stage								
T3	35	64.8	78.4	0.569	64.9	0.224	93.1	0.260
T4	19	35.2	79.3		82.5		100	
cN stage								
N0	2	3.7	50.0	0.336	50.0	0.540	100	0.162
N1	20	37.0	82.0		68.8		88.2	
N2	32	59.3	77.8		74.0		100	
Stage								
II	2	3.7	50.0	0.221	50.0	0.291	100	0.764
III	52	96.3	79.6		72.1		95.6	
CEA level								
Elevated	25	46.3	86.9	0.228	81.1	0.181	95.5	0.915
Normal	29	53.7	70.9		69.2		95.8	
CRM								
Positive	42	77.8	80.9	0.448	69.2	0.697	97.2	0.299
Negative	12	22.2	67.5		81.5		90.0	
EMVI								
Positive	38	70.4	80.0	0.655	77.0	0.766	97.1	0.488
Negative	16	29.6	78.7		69.7		92.3	
Down-stage T/mrTRG/								
Yes	33	61.1	81.8	0.108	81.2	0.019	96.7	0.666
No	21	38.9	73.7		55.0		94.4	
Down-stage N								
Yes	46	85.2	84.3	0.003	76.2	0.019	100	<0.001
No	8	14.8	50.0		42.9		71.4	
mrTRG								
Grade 1	5	9.3	100	0.027	80.0	0.017	100	0.030
Grade 2	37	68.5	85.3		76.6		100	
Grade 3	8	14.8	36.5		66.7		83.3	
Grade 4	4	7.4	50.0		25.0		66.7	
Grade 5	0	0	–		–		–	
pTRG								
Grade 0	12	22.2	71.4	0.798	70.1	0.291	100	0.002
Grade 1	10	18.5	88.9		88.9		100	
Grade 2	26	48.1	75.8		71.1		100	
Grade 3	6	11.1	83.3		50.0		66.7	

OS: overall survival, LRFS: local-regional recurrence-free survival, PFS: progression-free survival, cT stage: clinical T stage, cN stage: clinical N stage, EMVI: extramural venous invasion, CRM: circumferential resection margin, mrTRG: MRI-assessed tumor regression grade, pTRG: pathology-assessed tumor regression grade; CEA: carcinoembryonic antigen.

* The distance form anal verge assessed by MRI (Upper: >10cm; Middle: 5-10cm; Lower: ≤5cm).

"-" means unavailable.

Among the 54 patients, 33 patients received neo-CRT and surgery with postoperative ChT, and 21 patients received neo-CRT and surgery. The median RT doses were 50 Gy (range: 50-60 Gy) for GTVp and GTVn, and 45 Gy (range: 45-46 Gy) for CTV, all delivered in 25 daily fractions. The median cycle was 3 for neo-ChT (range: 1-7), and 4 for adjuvant ChT (range: 1-7). The total mesorectal excision (TME) was performed in most (53/54, 98.1%) patients after neo-CRT. The surgical procedures include Dixon (n=41), Miles (n=10), Parks (n=1), Hartmann(n=1), and local excision (n=1).

Of these 54 patients with complete mrTRG and pTRG assessment, 5 patients achieved mrTRG1 (9.3%), 37 achieved mrTRG2 (68.5%), 8 achieved mrTRG3 (14.8%), 4 achieved mrTRG4 (7.4%), and no patient achieved mrTRG5 after neo-CRT. One patient received R1 excision, and the remaining 53 patients received R0 excision. Regarding pTRG, 12 patients achieved pTRG0 (22.2%), 10 patients achieved pTRG1 (18.5%), 26 patients achieved pTRG2 (48.1%), and 6 patients achieved pTRG3 (11.1%) (**Table 3**).

**Table 3:** Comparison between mrTRG and pTRG.

		mrTRG					Total
		1	2	3	4	5	
**pTRG**	0	4	5	2	1	0	12
	1	0	7	2	1	0	10
	2	1	21	3	1	0	26
	3	0	4	1	1	0	6
**Total**		5	37	8	4	0	54

mrTRG, magnetic resonance tumor regression grade; pTRG, pathological tumour regression grade.

### The agreement between mrTRG and pTRG

The agreement between the three-tier mrTRG (mrTRG1 vs. mrTRG2-3 vs. mrTRG4-5) and pTRG (pTRG0 vs. pTRG1-2 vs. pTRG3) classes was fair (weighted kappa=0.287). In a dichotomous classification, assessment of the agreement between mrTRG(mrTRG1 vs. mrTRG2-5)and pTRG(pTRG0 vs. pTRG1-3) also resulted in fair agreement (weighted kappa =0.391).

When a dichotomous classification (mrTRG 1-2 vs. mrTRG 3-5) was used to assess the ability of mrTRG to predict PCR (pTRG0), 9 out of 12 patients (75.0%) were correctly identified. The sensitivity, specificity, and positive and negative predictive values of mrTRG were 75.0%, 21.4%, 21.4%, and 75.0%, respectively.

### Prognosis and survival

**Figure 4:**
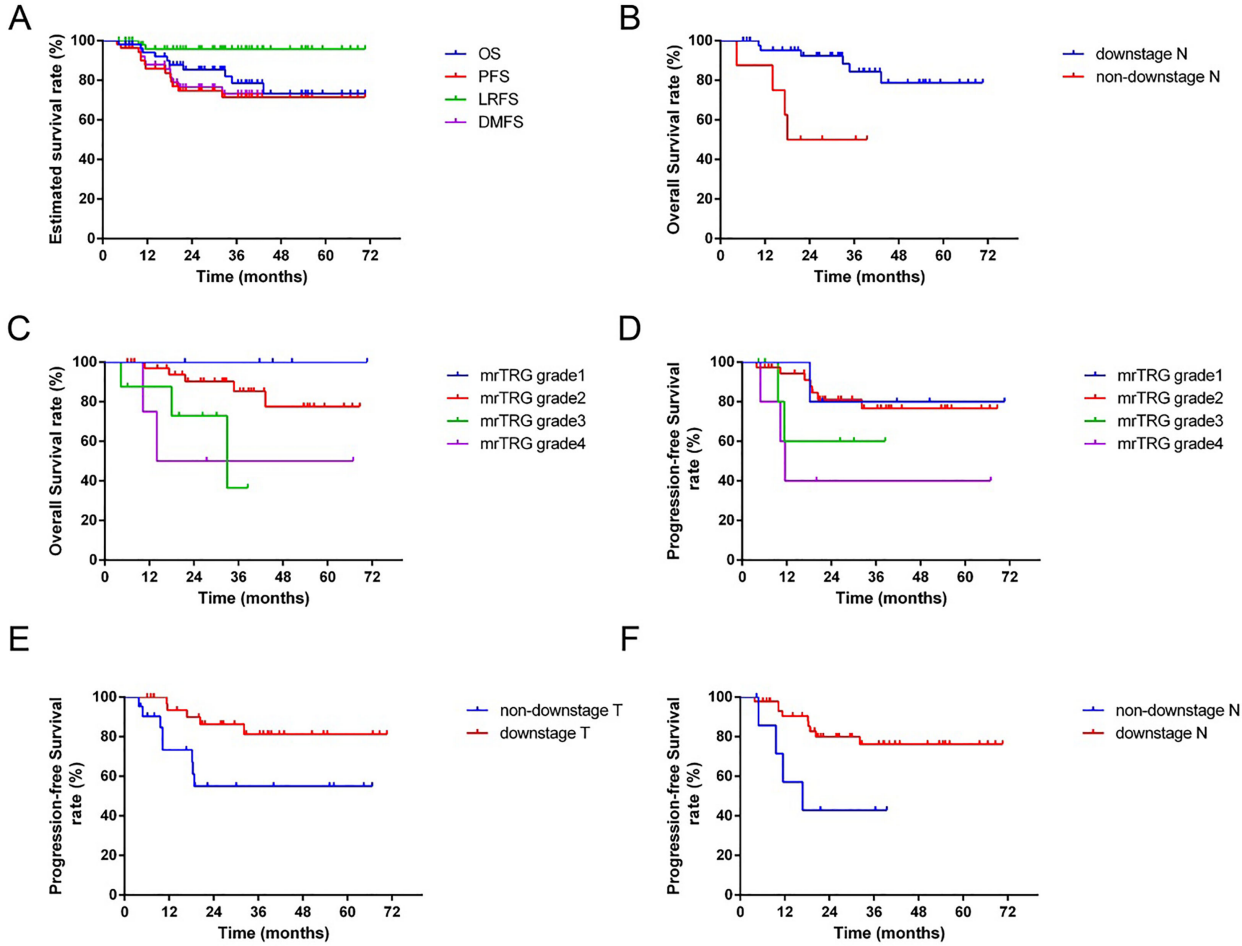
The survival curves for 54 patients. **(A)**: The estimated 3-year overall survival (OS), progress-free survival (PFS), local-regional recurrence-free survival (LRFS) and distance metastasis-free survival (DMFS) were 78.5%, 70.7%, 89.0%, and 75.2%, respectively. **(B)**: The 3-year OS of patients with downstage N was significantly better than the non-downstage N group (*p*=0.003). **(C)**: The 3-year OS of patients with favorable mrTRG was significantly better than the unfavorable group (*p*=0.027). **(D)**: The 3-year PFS of patients with favorable mrTRG was significantly better than the unfavorable group (*p*=0.017). **(E)**: The 3-year PFS of patients with downstage T was significantly better than the non-downstaging T group (*p*=0.019). **(F)**: The 3-year PFS of patients with downstage N was significantly better than the non-downstage N group (*p*=0.019).

The median follow-up time of all patients was 34.6 months (range: 4.4-70.1 months). The estimated 3-year OS, PFS, LRFS and DMFS were 78.5%, 70.7%, 89.0%, and 75.2%, respectively (**Figure 4A**).

During follow-up, two patients developed local-recurrence, and the tumor site was located anterior to the sacrum. Twelve patients developed distant metastases, and the most common metastatic sites included the lung (n=5), liver (n=3), retroperitoneal lymph nodes (n=3), and bone (n=1).

In univariate analysis, downstage N and favorable mrTRG were significantly associated with better OS (p=0.003 for downstage N, **Figure 4B**; *p*=0.027 for mrTRG, **Figure 4C**), while favorable mrTRG, downstage T and downstage N were significantly associated with better PFS (*p*=0.017 for mrTRG, **Figure 4D**; *p*=0.019 for downstage T, **Figure 4E**; *p*=0.019 for downstage N, **Figure 4F**) (**Table 2**). The factors with a *P* value of less than 0.1 in univariable analysis were included in the multivariable analysis. In multivariate analysis, downstaging N (*p*=0.045) was an independent prognostic factor for OS. Meanwhile, downstaging T (*p*=0.011) and downstaging N (*p*=0.012) were independent prognostic factors for PFS (**Table 4**).

**Table 4:** Multivariable Cox analysis of prognostic factors for OS and PFS.

Factors	OS		PFS	
	*P* value	HR (95%CI)	*P* value	HR (95%CI)
**Down-stage T**(Yes vs. No)	0.140	0.344 (0.084-1.417)	0.011	0.163 (0.040-0.665)
**Down-stage N**(Yes vs. No)	0.045	0.209 (0.045-0. 964)	0.012	0.153 (0.036-0.657)
**mrTRG** (Grade 1-2 vs. 3-4)	0.124	3.203 (0.727-14.118)	0.140	2.577 (0.733-9.059)
**pTRG** (PCR vs. non-PCR)	0.376	0.515 (0.118-2.240)	0.336	0.475 (0.104-2.164)

OS, Overall survival variable; PFS, Progress-free survival; HR, Hazard ratio; mrTRG, magnetic resonance tumour regression grade; pTRG, pathological tumour regression grade; PCR, pathological complete response.

## Discussion

The main finding of this research was that favorable mrTRG (mrTRG1-2) after neo-CRT could be used as a potential prognostic factor for LARC patients. Another valuable finding was that although the consistency between mrTRG and pTRG was fair, the sensitivity (75.0%) and negative predictive values (75.0%) of mrTRG 1-2 for PCR were satisfactory.

For LARC patients, neo-CRT followed by surgery and postoperative ChT was the standard care (2), and accurate restaging after neo-CRT and assessment of treatment response were critical to treatment decision-making throughout the process. In routine clinical work, MRI, postoperative pathological results, and hematologic tumor markers (e.g. CEA, CA199) are the most common detection means (19–23). However, there were some limitations in pathological and hematological markers. For example, postoperative pathological results can only be obtained after surgery, and hematology markers have certain fluctuations. With the advantages of high detection accuracy and noninvasiveness, pelvic MRI has become the most commonly used examination, for staging, restaging after neo-CRT and predicting the prognosis (4, 24). In this study, favorable mrTRG (mrTRG1-2) was significantly associated with better OS and PFS in univariate analysis, which was consistent with previous studies. However, pTRG (PCR vs. non-PCR) had no significant effect on prognosis in either univariate or multivariate analysis, which was different from previous research (8, 23, 25, 26). The main reason was that, compared with the PCR group (median cycle was 2, range: 0-5), patients in non-PCR group received more intense adjuvant ChT (median cycle was 4, range: 2-7). The different postoperative treatment strategies and small sample size of this study may reduce the difference in survival between the two groups.

In this study, the consistency between mrTRG and pTRG was fair (weighted kappa=0.287 for three-tier; weighted kappa=0.391 for a dichotomous classification), which was consistent with previous studies (12). However, the sensitivity (75.0%) and negative predictive values (75.0%) of mrTRG 1-2 for PCR were satisfactory. These results suggested that although mrTRG was not a surrogate of pTRG, favorable mrTRG may be a predictor for PCR. Therefore, in clinical work, patients with favorable mrTRG after neo-CRT could be recommended to adopt a “watch-and-wait” strategy, an organ preservation strategy to avoid complications from overtreatment, such as surgery. Previous study results showed no significant difference in recurrence and OS between patients managed with “watch-and-wait” after a clinical complete response and patients with PCR after operation (27).

Restaging MRI is more inclined to over stage of disease after neo-CRT in LARC patients as a result of the difficulties in assessing response within areas of post radiation fibrosis (28). Radiomics refers to the extraction of a vast number of qualitative and quantitative features from routine images using artificial intelligence that are effectively invisible to the human eye. MRI-based radiomics can help clinicians predict whether patients will achieve a PCR after neo-CRT before surgery to avoid excessive treatment (29). MRI-based radiomics has predictive value for the curative effect of neo-CRT on LARC patients and shows good predictive value in terms of tumor staging, postoperative metastasis, and prognosis after treatment (30). In the future, new MRI parameters could be added to mrTRG to increase the accuracy of PCR prediction and provide more valuable information to make treatment decisions.

There are some limitations to this study. First, the use of ultrasound gel is controversial and not recommended by the ESGAR guidelines. However, we filled the rectum with the appropriate amount of gel tailored to the size and location of the tumor. In this circumstance, rectal overdistension was avoided. Therefore, rectal gel filling had a minimal impact on the tumor staging evaluation. Moreover, rectal gel filling will reduce susceptibility artefacts related to luminal gas on DWI and may facilitate detection of smaller and treated tumor (31–33). Thus, rectal gel filling is useful for the mrTRG evaluation based on T2WI and DWI. Second, it was a retrospective study, and the surgical methods and ChT regimen were not completely uniform. Finally, the number of patients who met the criteria was small. In future work, we will produce a prospective study with large sample sizes to verify this result.

In conclusion, although the consistency between mrTRG and pTRG is only fair, favorable mrTRG after neo-CRT may be used as a potential prognostic factor for LARC patients’survival.

## Data availability statement

The raw data supporting the conclusions of this article will be made available by the authors, without undue reservation.

## Author contributions

SN, YC and FP drafted the manuscript and performed the statistical analysis; JW, JX, ZY and JP collected clinical data; YB and LD conceived the study and participated in its design and coordination. All authors contributed to the article and approved the submitted version.

## References

[1] Sung H Ferlay J Siegel RL Laversanne M Soerjomataram I Jemal A. Global cancer statistics 2020: GLOBOCAN estimates of incidence and mortality worldwide for 36 cancers in 185 countries. CA Cancer J Clin (2021) 71(3):209–49. doi: 10.3322/caac.21660 33538338

[2] Benson AB Venook AP Al-Hawary MM Azad N Chen YJ Ciombor KK. Rectal cancer, version 2.2022, NCCN clinical practice guidelines in oncology. J Natl Compr Canc Netw (2022) 20(10):1139–67. 10.6004/jnccn.2022.0051 36240850

[3] Kalisz KR Enzerra MD Paspulati RM. MRI Evaluation of the response of rectal cancer to neoadjuvant chemoradiation therapy. Radiographics (2019) 39(2):538–56. doi: 10.1148/rg.2019180075 30844347

[4] Taylor FG Quirke P Heald RJ Moran B Blomqvist L Swift I. Preoperative high-resolution magnetic resonance imaging can identify good prognosis stage I, II, and III rectal cancer best managed by surgery alone: a prospective, multicenter, European study. Ann Surg (2011) 253(4):711–9. doi: 10.1097/SLA.0b013e31820b8d52 21475011

[5] Patel UB Taylor F Blomqvist L George C Evans H Tekkis P. Magnetic resonance imaging-detected tumor response for locally advanced rectal cancer predicts survival outcomes: MERCURY experience. J Clin Oncol (2011) 29(28):3753–60. doi: 10.1200/JCO.2011.34.9068 21876084

[6] Fokas E Strobel P Fietkau R Ghadimi M Liersch T Grabenbauer GG. Tumor regression grading after preoperative chemoradiotherapy as a prognostic factor and individual-level surrogate for disease-free survival in rectal cancer. J Natl Cancer Inst (2017) 109(12). doi: 10.1093/jnci/djx095 29206996

[7] Dworak O Keilholz L Hoffmann A. Pathological features of rectal cancer after preoperative radiochemotherapy. Int J Colorectal Dis (1997) 12(1):19–23. doi: 10.1007/s003840050072 9112145

[8] Erlandsson J Lorinc E Ahlberg M Pettersson D Holm T Glimelius B. Tumour regression after radiotherapy for rectal cancer - results from the randomised Stockholm III trial. Radiother Oncol (2019) 135:178–86. doi: 10.1016/j.radonc.2019.03.016 31015165

[9] Mandard AM Dalibard F Mandard JC Marnay J Henry-Amar M Petiot JF. Pathologic assessment of tumor regression after preoperative chemoradiotherapy of esophageal carcinoma. clinicopathologic correlations. Cancer (1994) 73(11):2680–6. doi: 10.1002/1097-0142(19940601)73:11<2680::aid-cncr2820731105>3.0.co;2-c 8194005

[10] Jager T Neureiter D Urbas R Klieser E Hitzl W Emmanuel K. Applicability of American joint committee on cancer and college of American pathologists regression grading system in rectal cancer. Dis Colon Rectum (2017) 60(8):815–26. doi: 10.1097/DCR.0000000000000806 28682967

[11] Achilli P Magistro C Abd El Aziz MA Calini G Bertoglio CL Ferrari G. Modest agreement between magnetic resonance and pathological tumor regression after neoadjuvant therapy for rectal cancer in the real world. Int J Cancer (2022) 151(1):120–7. doi: 10.1002/ijc.33975 35191540

[12] Sclafani F Brown G Cunningham D Wotherspoon A Mendes LST Balyasnikova S. Comparison between MRI and pathology in the assessment of tumour regression grade in rectal cancer. Br J Cancer (2017) 117(10):1478–85. doi: 10.1038/bjc.2017.320 PMC5680467 28934761

[13] Smith JJ Strombom P Chow OS Roxburgh CS Lynn P Eaton A. Assessment of a watch-and-Wait strategy for rectal cancer in patients with a complete response after neoadjuvant therapy. JAMA Oncol (2019) 5(4):e185896. doi: 10.1001/jamaoncol.2018.5896 30629084 PMC6459120

[14] Myerson RJ Garofalo MC El Naqa I Abrams RA Apte A Bosch WR. Elective clinical target volumes for conformal therapy in anorectal cancer: a radiation therapy oncology group consensus panel contouring atlas. Int J Radiat Oncol Biol Phys (2009) 74(3):824–30. doi: 10.1016/j.ijrobp.2008.08.070 PMC2709288 19117696

[15] Jang JK Lee CM Park SH Kim JH Kim J Lim SB. How to combine diffusion-weighted and T2-weighted imaging for MRI assessment of pathologic complete response to neoadjuvant chemoradiotherapy in patients with rectal cancer? Korean J Radiol (2021) 22(9):1451–61. doi: 10.3348/kjr.2020.1403 PMC8390818 34132075

[16] Park MJ Kim SH Lee SJ Jang KM Rhim H. Locally advanced rectal cancer: added value of diffusion-weighted MR imaging for predicting tumor clearance of the mesorectal fascia after neoadjuvant chemotherapy and radiation therapy. Radiology (2011) 260(3):771–80. doi: 10.1148/radiol.11102135 21846762

[17] Ryan R Gibbons D Hyland JM Treanor D White A Mulcahy HE. Pathological response following long-course neoadjuvant chemoradiotherapy for locally advanced rectal cancer. Histopathology (2005) 47(2):141–6. doi: 10.1111/j.1365-2559.2005.02176.x 16045774

[18] Gavioli M Luppi G Losi L Bertolini F Santantonio M Falchi AM. Incidence and clinical impact of sterilized disease and minimal residual disease after preoperative radiochemotherapy for rectal cancer. Dis Colon Rectum (2005) 48(10):1851–7. doi: 10.1007/s10350-005-0133-6 16132481

[19] Horvat N Carlos Tavares Rocha C Clemente Oliveira B Petkovska I Gollub MJ. MRI Of rectal cancer: tumor staging, imaging techniques, and management. Radiographics (2019) 39(2):367–87. doi: 10.1148/rg.2019180114 PMC6438362 30768361

[20] Shin J Seo N Baek SE Son NH Lim JS Kim NK. MRI Radiomics model predicts pathologic complete response of rectal cancer following chemoradiotherapy. Radiology (2022) 303(2):351–8. doi: 10.1148/radiol.211986 35133200

[21] Wilson K Flood M Narasimhan V Pham T Warrier S Ramsay R. Complete pathological response in rectal cancer utilising novel treatment strategies for neo-adjuvant therapy: a systematic review. Eur J Surg Oncol (2021) 47(8):1862–74. doi: 10.1016/j.ejso.2021.03.245 33814240

[22] Kim JY Kim NK Sohn SK Kim YW Kim KJ Hur H. Prognostic value of postoperative CEA clearance in rectal cancer patients with high preoperative CEA levels. Ann Surg Oncol (2009) 16(10):2771–8. doi: 10.1245/s10434-009-0651-x PMC2749169 19657698

[23] Chen HY Feng LL Li M Ju HQ Ding Y Lan M. College of American pathologists tumor regression grading system for long-term outcome in patients with locally advanced rectal cancer. Oncologist (2021) 26(5):e780–93. doi: 10.1002/onco.13707 PMC8100558 33543577

[24] Taylor FG Quirke P Heald RJ Moran BJ Blomqvist L Swift IR. Magnetic resonance imaging in rectal cancer European equivalence study study G: preoperative magnetic resonance imaging assessment of circumferential resection margin predicts disease-free survival and local recurrence: 5-year follow-up results of the MERCURY study. J Clin Oncol (2014) 32(1):34–43. doi: 10.1200/JCO.2012.45.3258 24276776

[25] Sakin A Sahin S Sengul Samanci N Yasar N Demir C Geredeli C. The impact of tumor regression grade on long-term survival in locally advanced rectal cancer treated with preoperative chemoradiotherapy. J Oncol Pharm Pract (2020) 26(7):1611–20. doi: 10.1177/1078155219900944 32046577

[26] Fanelli GN Loupakis F Smyth E Scarpa M Lonardi S Pucciarelli S. Pathological tumor regression grade classifications in gastrointestinal cancers: role on patients' prognosis. Int J Surg Pathol (2019) 27(8):816–35. doi: 10.1177/1066896919869477 31416371

[27] Dossa F Chesney TR Acuna SA Baxter NN. A watch-and-wait approach for locally advanced rectal cancer after a clinical complete response following neoadjuvant chemoradiation: a systematic review and meta-analysis. Lancet Gastroenterol Hepatol (2017) 2(7):501–13. doi: 10.1016/S2468-1253(17)30074-2 28479372

[28] Jia X Zhang Y Wang Y Feng C Shen D Ye Y. MRI For restaging locally advanced rectal cancer: detailed analysis of discrepancies with the pathologic reference standard. AJR Am J Roentgenol (2019) 213(5):1081–90. doi: 10.2214/AJR.19.21383 31386575

[29] Lambregts DMJ Boellaard TN Beets-Tan RGH. Response evaluation after neoadjuvant treatment for rectal cancer using modern MR imaging: a pictorial review. Insights Imaging (2019) 10(1):15. doi: 10.1186/s13244-019-0706-x 30758688 PMC6375095

[30] Qin Y Zhu LH Zhao W Wang JJ Wang H. Review of radiomics- and dosiomics-based predicting models for rectal cancer. Front Oncol (2022) 12:913683. doi: 10.3389/fonc.2022.913683 36016617 PMC9395725

[31] Beets-Tan RGH Lambregts DMJ Maas M Bipat S Barbaro B Curvo-Semedo L. Magnetic resonance imaging for clinical management of rectal cancer: updated recommendations from the 2016 European society of gastrointestinal and abdominal radiology (ESGAR) consensus meeting. Eur Radiol (2018) 28(4):1465–75. doi: 10.1007/s00330-017-5026-2 PMC5834554 29043428

[32] Nougaret S Reinhold C Mikhael HW Rouanet P Bibeau F Brown G. The use of MR imaging in treatment planning for patients with rectal carcinoma: have you checked the "DISTANCE"? Radiology (2013) 268(2):330–44. doi: 10.1148/radiol.13121361 23882096

[33] Kaur H Choi H You YN Rauch GM Jensen CT Hou P. MR imaging for preoperative evaluation of primary rectal cancer: practical considerations. Radiographics (2012) 32(2):389–409. doi: 10.1148/rg.322115122 22411939

