# Supplementary material for: The role of MRI after neochemoradiotherapy in predicting pathological tumor regression grade and clinical outcome in patients with locally advanced rectal adenocarcinoma
Source: Front Oncol. 2023 Jun 12;13:1118518. doi: 10.3389/fonc.2023.1118518 (PMC10292078; doi:10.3389/fonc.2023.1118518)
Supplement: Supplementary file 1 [file Table_1.docx]

**Supplementary Material**

**Supplementary Figures and Tables**

Table 1. The MRI Tumor Regression Grade (mrTRG) evaluation criteria adopted in this study^[1]^.

| **MRI Tumor Regression Grade** | **Definition of Grade** |
| --- | --- |
| Grade 1:  Complete radiologic response | No evidence of tumor |
| Grade 2:  Good response | Dense (>75%) fibrosis with no obvious residual tumor |
| Grade 3:  Moderate response | >50% fibrosis or mucin with a minority of visible tumor |
| Grade 4:  Slight response | <50% fibrosis or mucin with a majority of visible tumor |
| Grade 5:  No response | No posttreatment changes (same as before treatment) |

Table 2. The pathological grade tumor response (pTRG) assessment system recommended by the AJCC Cancer Staging Manual, Eighth Edition and the CAP Guidelines ^[2, 3]^ was adopted in this study.

| **Pathological Tumor**  **Regression Grade** | **Definition of Grade** |
| --- | --- |
| Grade 0：Complete response | No remaining viable cancer cells |
| Grade 1：Moderate response | Only small clusters or single cancer cells remaining |
| Grade 2：Minimal response | Residual cancer remaining, but with predominant fibrosis |
| Grade 3：Poor response | Minimal or no tumor kill; extensive residual cancer |

**Abbreviation**: mrTRG, magnetic resonance tumour regression grade; pTRG，pathological tumour regression grade.

**References:**

1. Kalisz KR, Enzerra MD, Paspulati RM: **MRI Evaluation of the Response of Rectal Cancer to Neoadjuvant Chemoradiation Therapy**. *Radiographics* 2019, **39**(2):538-556.

2. Ryan R, Gibbons D, Hyland JM, Treanor D, White A, Mulcahy HE, O'Donoghue DP, Moriarty M, Fennelly D, Sheahan K: **Pathological response following long-course neoadjuvant chemoradiotherapy for locally advanced rectal cancer**. *Histopathology* 2005, **47**(2):141-146.

3. Gavioli M, Luppi G, Losi L, Bertolini F, Santantonio M, Falchi AM, D'Amico R, Conte PF, Natalini G: **Incidence and clinical impact of sterilized disease and minimal residual disease after preoperative radiochemotherapy for rectal cancer**. *Dis Colon Rectum* 2005, **48**(10):1851-1857.
